# Kiss-and-Run Is a Significant Contributor to Synaptic Exocytosis and Endocytosis in Photoreceptors

**DOI:** 10.3389/fncel.2017.00286

**Published:** 2017-09-20

**Authors:** Xiangyi Wen, Grant W. Saltzgaber, Wallace B. Thoreson

**Affiliations:** ^1^Department of Pharmacology & Experimental Neuroscience, University of Nebraska Medical Center Omaha, NE, United States; ^2^Ophthalmology & Visual Sciences, Truhlsen Eye Institute, University of Nebraska Medical Center Omaha, NE, United States

**Keywords:** kiss-and-run, endocytosis, exocytosis, photoreceptor cells, vertebrate, ribbon synapses

## Abstract

Accompanying sustained release in darkness, rod and cone photoreceptors exhibit rapid endocytosis of synaptic vesicles. Membrane capacitance measurements indicated that rapid endocytosis retrieves at least 70% of the exocytotic membrane increase. One mechanism for rapid endocytosis is kiss-and-run fusion where vesicles briefly contact the plasma membrane through a small fusion pore. Release can also occur by full-collapse in which vesicles merge completely with the plasma membrane. We assessed relative contributions of full-collapse and kiss-and-run in salamander photoreceptors using optical techniques to measure endocytosis and exocytosis of large vs. small dye molecules. Incubation with small dyes (SR101, 1 nm; 3-kDa dextran-conjugated Texas Red, 2.3 nm) loaded rod and cone synaptic terminals much more readily than larger dyes (10-kDa Texas Red, 4.6 nm; 10-kDa pHrodo, 4.6 nm; 70-kDa Texas Red, 12 nm) consistent with significant uptake through 2.3–4.6 nm fusion pores. By using total internal reflection fluorescence microscopy (TIRFM) to image individual vesicles, when rods were incubated simultaneously with Texas Red and AlexaFluor-488 dyes conjugated to either 3-kDa or 10-kDa dextran, more vesicles loaded small molecules than large molecules. Using TIRFM to detect release by the disappearance of dye-loaded vesicles, we found that SR101 and 3-kDa Texas Red were released from individual vesicles more readily than 10-kDa and 70-kDa Texas Red. Although 10-kDa pHrodo was endocytosed poorly like other large dyes, the fraction of release events was similar to SR101 and 3-kDa Texas Red. We hypothesize that while 10-kDa pHrodo may not exit through a fusion pore, release of intravesicular protons can promote detection of fusion events by rapidly quenching fluorescence of this pH-sensitive dye. Assuming that large molecules can only be released by full-collapse whereas small molecules can be released by both modes, our results indicate that 50%–70% of release from rods involves kiss-and-run with 2.3–4.6 nm fusion pores. Rapid retrieval of vesicles by kiss-and-run may limit membrane disruption of release site function during ongoing release at photoreceptor ribbon synapses.

## Introduction

Vertebrate photoreceptors maintain a relatively depolarized membrane potential in darkness near −40 mV, allowing the continuous release of glutamate-filled vesicles at ribbon-style synapses. Photoreceptors do not generate sodium-dependent action potentials but instead respond to light with graded changes in membrane potential. Release slows when photoreceptors hyperpolarize to light. To assist with continuous release, photoreceptors possess a large vesicle pool and a specialized complement of synaptic proteins (Morgans et al., 1996; Schmitz et al., 2000; Thoreson et al., 2004; Reim et al., 2005; Magupalli et al., 2008; Duncan et al., 2010). However, even the large pool of 216,000 vesicles in a salamander cone synapse would be emptied in <15 min by sustained release in darkness if not replenished by endocytosis (Sheng et al., 2007). Mechanisms for rapid and robust endocytic retrieval are therefore essential to maintain continuous release.

Exocytosis and endocytosis of synaptic vesicles can be coupled in two general ways (Alabi and Tsien, 2013; Wu et al., 2014). In the full-collapse model, the vesicle membrane merges completely with the plasma membrane and vesicles must be fully reconstructed and retrieved during endocytosis. In the kiss-and-run model, a vesicle briefly contacts the plasma membrane through a small fusion pore that permits release of small molecules but the vesicle does not flatten into the plasma membrane. The vesicle with its complement of proteins is quickly recycled to the cytoplasm after closure of the fusion pore. By speeding vesicle recycling, kiss-and-run can provide an efficient mechanism to facilitate sustained release.

Kiss-and-run fusion events have been observed in both neurons and neuroendocrine cells (Albillos et al., 1997; Holroyd et al., 2002; Wang et al., 2003; Fulop and Smith, 2006; He et al., 2006; Vardjan et al., 2007). In neuroendocrine cells, kiss-and-run allows selective release of small molecules while retaining larger peptides (Liang et al., 2017). The fraction of release events that arise from kiss-and-run in neurons may vary with experimental preparations and conditions. Some studies have suggested that kiss-and-run in neurons is quite rare (Zenisek et al., 2002; Granseth et al., 2006; He et al., 2006; Balaji and Ryan, 2007; Chen et al., 2008) while others have suggested that it can contribute to a majority of release events in certain preparations or under certain conditions (Gandhi and Stevens, 2003; Richards et al., 2005; Photowala et al., 2006; Zhang et al., 2009).

Characteristics used to distinguish kiss-and-run from full-collapse fusion include rapid endocytic kinetics and the small size of the fusion pore formed during kiss-and-run. Whole-cell capacitance measurements showed that vesicles can be retrieved in rods and cones by a rapid form of endocytosis (Van Hook and Thoreson, 2012; Cork and Thoreson, 2014). Although rapid endocytosis is consistent with possible contributions from kiss-and-run, it can also arise from other mechanisms (Watanabe et al., 2013; Watanabe and Boucrot, 2017). To assess the frequency of kiss-and-run and full-collapse events in salamander rods and cones, we measured both exocytosis and endocytosis of synaptic vesicles using multiple fluorescent dyes ranging from 1 nm to 12 nm diameter (Takahashi et al., 2002). Use of optical techniques allowed us to measure the properties of release and retrieval in many vesicles. We assessed endocytosis by measuring changes in whole-terminal fluorescence and by imaging individual vesicles using electron microscopy and total internal reflection fluorescence microscopy (TIRFM). We measured exocytosis by using TIRFM to image release of individual vesicles. We also used membrane capacitance measurements to assess contributions from rapid and slow modes of endocytosis. We found that small molecules (≤2.3 nm) were both released and retrieved much more readily than large molecules (≥4.6 nm). Together with capacitance measurements, these results suggest that kiss-and-run events with a fusion pore diameter between 2.3 and 4.6 nm contribute to at least 50% of vesicle release and recycling in photoreceptor terminals. By limiting possible disruption of neighboring release sites along the ribbon base that can be produced by prior fusion events, rapid retrieval by kiss-and-run may be particularly useful for maintaining release at continuously active photoreceptor ribbon synapses.

## Materials and Methods

### Animal Care and Use

Aquatic tiger salamanders (*Ambystoma tigrinum*, 18–25 cm; Sullivan Company, Nashville, TN, USA) were maintained on a 12-h light/dark cycle and sacrificed after ≥1 h of dark adaptation. Salamanders were anesthetized by immersion in 0.25 g/L tricaine methanesulfonate (Western Chemical) for >15 min, decapitated with heavy shears, and pithed. Procedures were approved by the University of Nebraska Medical Center Institutional Animal Care and Use Committee.

### Reagents

Unless otherwise noted, reagents were from Sigma-Aldrich Chemicals.

### Photoreceptor Isolation

After enucleation and removal of the anterior segment, the eyecup was cut in half and the retina isolated in Ca^2+^-free, high-Mg^2+^ amphibian saline consisting of (in mM): 116 NaCl, 2.5 KCl, 5 MgCl_2_, 5 glucose, and 10 HEPES (pH 7.4). Retinal pieces were incubated with 30 units/ml papain (Worthington) activated with 0.2 mg/ml cysteine in Ca^2+^-free high-Mg^2+^ saline solution for 35 min at room temperature. Afterwards, retinal pieces were transferred to ice-cold Ca^2+^-free, high-Mg^2+^ saline with 1% bovine serum albumin for 3 min. Retinal pieces were washed another 3 min in ice-cold Ca^2+^-free, high-Mg^2+^ saline and then incubated in ice-cold Ca^2+^-free, high-Mg^2+^ saline containing DNase (4000 μ/ml; Worthington) for another 5 min. Photoreceptors were isolated by gently triturating the tissue ~10 times through the tip of a fire-polished, bent Pasteur pipette.

Isolated cells were plated on glass coverslips with a refractive index of 1.52 (#1 Coverslips, Warner Instruments) or 1.78 (Olympus) and coated with 3.5 μg/cm^2^ Cell-Tak (BD Biosciences).

### Capacitance Measurements of Endocytosis

Whole-cell recordings for capacitance measurements were obtained from isolated photoreceptors and photoreceptors in retinal slices. For electrophysiological recording, isolated cells were plated on glass slides coated with 1 mg/ml concanavalin A.

Retinal slice preparation has been detailed elsewhere (Van Hook and Thoreson, 2012). Briefly, a piece of eyecup was placed vitreal surface down onto nitrocellulose membrane (5 × 10 mm, 0.8 μm pores; Millipore), the retina was isolated, and then cut into 125 μm thick slices using a razor blade tissue slicer (Stoelting). Slices were viewed through an upright fixed-stage microscope (Olympus BH2-WI) and superfused at room temperature at ~1 ml min^−1^ with oxygenated amphibian saline containing (in mM): 116 NaCl, 2.5 KCl, 1.8 CaCl_2_, 0.5 MgCl_2_, 5 glucose, and 10 HEPES (pH 7.8).

Whole-cell capacitance measurements were described previously (Van Hook and Thoreson, 2012). Borosilicate glass pipettes (1.2 mm OD, 0.9 mm ID, with internal filament; World Precision Instruments) were pulled with a vertical pipette puller (Narishige). Pipette shafts were coated with dental wax. Pipettes were filled with (in mM): 50 CsGluconate, 40 CsGlutamate, 10 TEACl, 3.5 NaCl, 1 CaCl_2_, 1 MgCl_2_, 9.4 MgATP, 0.5 GTP-Na, 5 EGTA (pH 7.2). Membrane capacitance was measured with phase tracking hardware integrated into the Optopatch amplifier (Cairn Research), acquired with Clampex 10.4 software (Molecular Devices), and analyzed with Clampfit 10.4 software. Series resistance (R_s_), membrane resistance (R_m_), and capacitance (C_m_) averaged: 29.6 ± 1.5 MΩ (R_s_), 200.3 ± 20.4 MΩ (R_m_) and 58.7 ± 2.9 pF (C_m_) for cones (*N* = 7) in slices; 35.0 ± 4.1 MΩ, 225.5 ± 48.2 MΩ, and 23.5 ± 1.6 pF for rods (*N* = 6) in slices; 38.0 ± 5.1 MΩ, 1102.0 ± 238.8 MΩ, and 15.1 ± 0.7 pF for isolated cones (*N* = 7); and 34.5 ± 3.4 MΩ, 410.3 ± 80.4 MΩ and 15.1 ± 1.3 pF for isolated rods (*N* = 12). Charging curves of rods and cones are well fit by single exponentials indicating a compact electrotonic structure (Van Hook and Thoreson, 2012). Responses were excluded if holding currents exceeded 250 pA, access resistance exceeded 50 MΩ, or if there were large changes in R_s_ during the test step. Photoreceptors were depolarized with a 25 ms step from −70 mV to −10 mV. Capacitance measurements were begun after tail currents had subsided completely, typically ~250 ms after terminating the test step. Rates of endocytosis were determined by fitting capacitance declines with a single exponential function.

### Whole-Terminal Fluorescence Measurements

After letting cells settle onto coverslips, isolated photoreceptors were incubated with SR101, 3-, 10-, 70-kDa dextran-conjugated Texas Red, or 10-kDa dextran-conjugated pHrodo (Molecular Probes, Invitrogen, 7 μM) in amphibian saline for 3 or 10 min. Basal release was measured by incubating photoreceptors for 10 min with dye in Ca^2+^-free, high-Mg^2+^ solution containing 0.1 mM Cd^2+^. Cells were superfused with oxygenated amphibian saline for at least 10 min before measurements.

In experiments with dynasore (Abcam) and pitstop-2, retinal pieces were pre-treated with drug in Ca^2+^-free high-Mg^2+^ saline for 20 min and then transferred to a solution containing dye (67 μM 3-kDa Texas Red or 50 μM 10-kDa Texas Red) and drug for 10 min. Photoreceptors were isolated and plated after dye loading.

Whole-terminal fluorescence was measured on an inverted microscope (Olympus IX71) through a 1.45 NA/60×, oil-immersion objective. Fluorescence emission was collected with 40-ms exposure times by an EMCCD camera (Hamamatsu ImageEM) through a 609 nm (54 nm wide) bandpass filter (Semrock). Background fluorescence was measured in adjacent regions outside the cell and subtracted from measurements of terminal fluorescence. Data were acquired and analyzed using MetaMorph software (Molecular Devices).

### *In Vitro* Dye Fluorescence Measurements

To compare intraterminal fluorescence measured with different dyes, the brightness of each dye was measured *in vitro* at three points along the shaft of a dye-filled patch pipette (0.25 NA/10× objective; Olympus). The molar fluorescent brightness (*F’*/μM) for each dye was: SR101: 25,040 arbitrary fluorescence units (a.u.)/μM, 3-kDa dextran-conjugated Texas Red: 2,084 a.u./μM, 10-kDa Texas Red: 10,716 a.u./μM, 70-kDa Texas Red: 22,837 a.u./μM, and pHrodo: 12,882 a.u./μM (pH = 5.5); 6901 a.u./μM (pH = 7.8). Dextran-conjugated 3-, 10-, 70-kDa Texas Red are sulfonyl derivatives of SR101. Dextran-conjugated 10-kDa pHrodo is a pH sensitive form of rhodamine that is brighter in acidic environments. Fluorescence of pHrodo was measured at both pH 5.5 and 7.8. To calculate relative fluorescence values during whole terminal measurements of endocytosis, we used pHrodo fluorescence measured at pH 5.5.

### Dye Release Measurements in Retinal Slices

For studies of release from retinal slices, pieces of isolated retina were loaded by incubation in darkness with SR101 (83 μM, 30 min), 3-kDa Texas Red (133 μM, 45 min), 10-kDa Texas Red (100 μM, 60 min), or 70-kDa Texas Red (36 μM, 60 min). After incubation, retina pieces were washed in Ca^2+^-free, high-Mg^2+^ saline and slices were prepared as described above. The recording chamber was placed on an upright fixed-stage microscope (Nikon E600 FN) equipped with a spinning disk laser confocal scan head (PerkinElmer UltraView LCI). Dyes were excited at 568 nm with an Ar/Kr laser. Emitted light was captured through 600 nm interference filters by a cooled CCD camera (Hamamatsu OrcaER). Excitation and emission were controlled by a Lambda 10-2 filter wheel and controller (Sutter Instrument). Images were acquired once per minute and analyzed with NIS-Elements AR software (Nikon). After collecting baseline data for 5 min, release was stimulated by applying 50 mM KCl saline (in mM: 68.6 NaCl, 50 KCl, 1.8 CaCl_2_, 0.5 MgCl_2_, 5 glucose, and 10 HEPES, pH 7.8) for another 15 min.

### Electron Microscopy

For electron microscopy, retinal pieces were loaded with 3- or 10-kDa Texas Red (7 μM, lysine fixable) in Ca^2+^-free, high-Mg^2+^ saline for 2 min and then incubated in a solution containing 1.8 mM Ca^2+^ and 20 mM K^+^ saline with the same dyes for eight additional minutes. Retinal pieces were then washed with Ca^2+^-free, high-Mg^2+^ saline three times for 3 min apiece. Retinal pieces were fixed overnight at 4°C in 2% glutaraldehyde, 2% paraformaldehyde, and 0.1 M Sorensen’s phosphate buffer (pH = 7.4). After fixation, retinas were washed in 0.1 M Tris buffer (pH = 7.6) for 2 min and then incubated in 0.01% 3,3′-diaminobenzidine tetrahydrochloride (DAB) in 0.1 M Tris buffer for 10 min. Dyes were photoconverted for 30 min in the presence of DAB (Maranto, 1982; Schmued and Snavely, 1993) by 609 nm light through a 0.25 NA, 10× objective. After photoconversion, retinal pieces were washed twice in phosphate-buffered saline and then placed in 1% osmium tetroxide. Samples were dehydrated through a graded ethanol series, followed by three washes with 100% propylene oxide. Samples were left overnight in a 1:1 mixture of Araldite embedding medium and propylene oxide, embedded in fresh Araldite in silicon rubber molds, and then placed in an oven at 65°C overnight. Resulting blocks were thin sectioned on a Leica UC6 ultramicrotome and placed on 200 mesh copper grids. Sections were stained with 1% uranyl acetate and Reynold’s lead citrate. Sections were examined in a FEI Tecnai G2 TEM operated at 80 kV.

### TIRFM Experiments

Individual synaptic vesicles were visualized in rod terminals using TIRFM as described previously (Chen et al., 2013). Solid-state lasers (561 nm or 488 nm; Melles Griot) were focused off-axis through the objective (100×, 1.65 N.A., oil immersion, Olympus) so that the beam underwent total internal reflection at the interface between the coverslip and overlying cell membrane or aqueous solution. We used an incident angle of ~60° that generates an evanescent wave with this objective exhibiting length constants of ~65 nm and ~57 nm for 561 nm and 488 nm lasers, respectively. Fluorescent emission was collected through 609 nm bandpass (54 nm wide, Semrock) or 500–545 nm/575–710 nm bandpass (Chroma) filters using an EMCCD camera (Hamamatsu ImageEM) at 40 ms/frame with a pixel size of 80 nm/pixel. TIRFM data were acquired using MetaMorph software and analyzed using MetaMorph and ImageJ.

To load dye for TIRFM experiments on release, tissue was dissected and retinas were isolated in darkness using GenIII image intensifiers (Nitemate NAV3, Litton Industries) mounted on a dissecting microscope. Maintaining retinae in a dark-adapted state keeps photoreceptors at a resting membrane potential of approximately −40 mV and thus promotes vesicle recycling. Retinal pieces were typically incubated in dye for a short time to load only a small percentage of vesicles: SR101 (8.3 μM, 1.5 min), 3-kDa Texas Red (133.3 μM, 1.5 min), 10-kDa Texas Red (50 μM, 3 min), and pHrodo (50 μM, 3 min). We incubated tissue with 70-kDa Texas Red (14.3 μM) for 30 min because it loaded quite poorly.

Synaptic vesicle release was stimulated by depolarizing rod terminals with a 2-s puff of 50 mM KCl delivered from a glass patch pipette using a pressure valve system (Toohey Company; 8 psi). The pipette tip was positioned 10–20 μm from rod terminals. For TIRFM experiments on endocytosis we loaded red and green dyes simultaneously in various combinations—3- or 10-kDa Texas Red (7 μM, zwitterionic) with 3- or 10-kDa AlexaFluor-488 (7 μM, anionic)—by incubating pieces of whole retina in dye solution prior to cell isolation. When we incubated isolated cells in dye after plating, many dye molecules adhered to coverslips, making it difficult to distinguish dye molecules within synaptic vesicles from extracellular dye molecules stuck to the glass. To limit vesicle cycling as dyes diffused through the retina, we first loaded the two dyes in Ca^2+^-free, high-Mg^2+^ saline for 2 min. To further limit vesicle cycling during this period, we also strongly light-adapted retinas by illuminating them with bright white light for 10 min before dye incubation. As a basal measurement of endocytosis in Ca^2+^-free conditions, we loaded one sample of retinas for another 2 min (for a total of 4 min) in Ca^2+^-free, high-Mg^2+^ saline. In a second sample, we followed the first 2 min of incubation in Ca^2+^-free, high-Mg^2+^ saline with 2 min incubation in a solution containing 1.8 mM Ca^2+^ and 20 mM K^+^ saline. After isolating and plating, rods were examined in the presence of Cd^2+^ (0.1 mM) to block Ca^2+^ channels and limit further vesicle cycling. Excitation was alternated between 561 nm and 488 nm (30 frames apiece, 40-ms/ frame) at 140-ms intervals. We used ImageJ to identify the terminal footprint and local maxima in average TIRFM images that were used to count the number of dye-loaded vesicles.

For release experiments, only vesicles with a signal-to-noise ratio >4:1 in a 7 × 7 pixel region of interest were analyzed. As described in the results, we compared the rise in fluorescence as vesicles approached the membrane to rates of fluorescence decline to distinguish release events from departure of vesicles from the membrane without release.

### Statistical Analysis

Unless otherwise noted, data were analyzed by Prism 4.0 (GraphPad Software) and results are presented as mean ± standard error of the mean (SEM).

## Results

### Rapid Endocytosis in Photoreceptors

Whole-cell capacitance measurements suggested the presence of rapid endocytosis in rod and cone photoreceptors (Van Hook and Thoreson, 2012; Cork and Thoreson, 2014). However, we were concerned that the time course of endocytosis in these earlier studies may have been influenced by I_h_ currents or by anion currents associated with the activity of presynaptic glutamate transporters. To address this, we measured exocytotic capacitance changes while bath applying CsCl (3 mM) to block I_h_ and DL-TBOA (100 μM) to block glutamate transporters. We measured capacitance changes in rods and cones from retinal slices evoked by depolarizing steps (−70 mV to −10 mV, 25 ms) to stimulate exocytosis. Step-evoked capacitance jumps averaged 80.1 ± 11.9 fF in cones (*N* = 10) and 54.3 ± 18.6 fF (*N* = 6) in rods. Non-ribbon release from rods was minimized by using brief 25 ms steps (Chen et al., 2013). Consistent with a synaptic origin, we observed paired pulse depression of capacitance jumps (Rabl et al., 2006). Other evidence that depolarization-evoked capacitance responses in salamander rods and cones derive from synaptic release include matches between the amplitude and kinetics of capacitance changes and the amplitude and kinetics of synaptic currents measured in paired photoreceptor/horizontal cell recordings (Thoreson et al., 2004; Rabl et al., 2005). Exocytotic capacitance jumps also ran down more quickly than calcium-activated chloride tail currents (Thoreson et al., 2004; Rabl et al., 2005; Van Hook and Thoreson, 2013; Cork and Thoreson, 2014).

**Figure 1:**
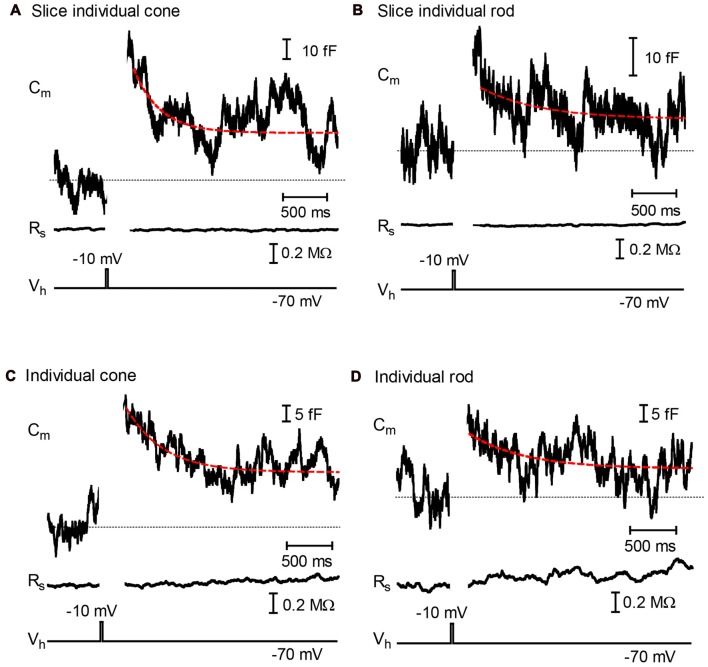
Fast endocytosis in photoreceptors. Whole-cell capacitance measured in photoreceptors from retinal slices **(A,B)** and isolated cells **(C,D)** in the presence of DL-TBOA (100 μM) and CsCl (3 mM). **(A,B)** show responses from a cone and rod, respectively, measured in retinal slices. **(C,D)** show responses from an isolated cone and rod, respectively. Depolarization (−70 mV to −10 mV, 25 ms) stimulated an increase in membrane capacitance that subsequently returned to baseline due to endocytosis. Endocytic capacitance decays were fit by single exponential curves (red lines): **(A)** tau = 297.8 ms, amplitude (a) = 0.0320308 pF, plateau amplitude (c) = 0.026172 pF; **(B)** tau = 608.3, *a* = 0.0081128, *c* = −0.43617; **(C)** tau = 602.7, *a* = 0.028183, *c* = 0.011441; **(D)** tau = 599.9, *a* = 0.010456, *c* = 0.099100. Dashed line shows the initial baseline of each trace.

The rate of endocytic membrane retrieval was assessed by fitting declines in membrane capacitance with a single exponential curve (Figure 1). To avoid possible contamination by membrane conductance changes, fitting was begun after the end of any tail currents, typically ~250 ms after termination of the step. As illustrated in Figures 1A,B, depolarization-evoked jumps in capacitance showed an initial decline with time constants of 570 ± 109 ms in cones (*N* = 10) and 492 ± 78 ms (*N* = 6) in rods. After this initial rapid phase of retrieval, membrane capacitance often leveled out above baseline. This suggests the presence of an additional slower component of endocytosis similar to that observed previously in salamander cones (Innocenti and Heidelberger, 2008). In cones, particularly in retinal slices, membrane capacitance sometimes overshot baseline, consistent with the excess endocytosis observed by Van Hook and Thoreson (2012). During the 5 s trial, the initial rapid phase of endocytosis recovered 78.8 ± 5.3% and 67.6 ± 7.6% of the exocytotic capacitance increase in cones and rods from retinal slices, respectively.

We further minimized the potential impact of glutamate transporter activity by measuring capacitance responses in the presence of TBOA and Cs^+^ using enzymatically-isolated cones and rods. Capacitance jumps evoked by 25 ms steps averaged 26.7 ± 4.0 fF in isolated cones (*N* = 7) and 53.3 ± 8.6 fF in isolated rods (*N* = 12). As illustrated in Figure 1, rates of endocytosis in isolated rods and cones were similar to those observed in retinal slices. Endocytosis in rods and cones showed an initial rapid decline that recovered 66.3 ± 7.2% and 71.5 ± 6.4% of the exocytotic capacitance increase, respectively, and exhibited time constants averaging 649 ± 86 ms and 672 ± 142 ms. In addition to showing that glutamate transporter activity is unlikely to be responsible for the fast capacitance decline, measurements from isolated cells indicate that enzymatic isolation and plating of photoreceptors did not greatly distort their synaptic function. The presence of rapid endocytosis is consistent with significant contributions from kiss-and-run fusion, but it can also be explained by other forms of rapid retrieval (Watanabe et al., 2013). We therefore turned to optical methods to help distinguish contributions of kiss-and-run from full-collapse fusion.

### Small Dyes Were Loaded Preferentially into Synaptic Terminals

Because kiss-and-run involves smaller fusion pores than full-collapse fusion, we made optical measurements of exocytosis and endocytosis using a range of different size dyes. We used SR101 and its sulfonyl derivative Texas Red conjugated to dextrans of 3-, 10- and 70-kDa (7 μM) with Stokes’ diameters of 1, 2.3, 4.6 and 12 nm, respectively (Armstrong et al., 2004; Erickson, 2009; Choi et al., 2010)^1^. We also tested 10-kDa dextran conjugated to a pH-sensitive dye, pHrodo.

**Figure 2:**
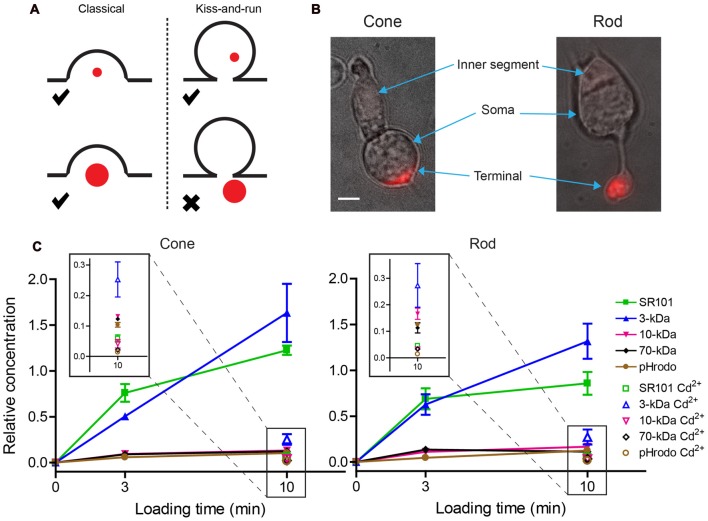
Small dyes were loaded preferentially into photoreceptor terminals over large dyes. **(A)** Schematic image illustrating the idea that small dyes can be endocytosed by both classical and kiss-and-run endocytosis, whereas large dyes can be engulfed by classical endocytosis but would be rejected by kiss-and-run. **(B)** Examples of an isolated salamander cone and rod loaded by incubation with SR101 (7 μM, 3 min). Inner segments, cell somas, and brightly fluorescent synaptic terminals are indicated by arrows. Scale bar: 5 μm. **(C)** SR101, 3-kDa Texas Red, 10-kDa Texas Red, 70-kDa Texas Red, and 10-kDa pHrodo (7 μM) were loaded by incubating isolated cones and rods for 3 or 10 min. Basal loading was measured by incubating cells for 10 min in Ca^2+^-free, high-Mg^2+^ saline solution with 0.1 mM Cd^2+^. Specific brightness of each dye (*F’*) relative to its concentration was measured *in vitro*. The relative intra-terminal concentration for each dye was calculated as follows: *Relative concentration* = $\frac{F}{F\prime /\mu M}$ where *F* is whole terminal fluorescence. Insets show data measured after 10-min loading at low relative concentration. Each data point is the average ± standard error of the mean (SEM) from three replicates. For each replicate, we measured intraterminal fluorescence from 5 to 70 cells (average of 48 cones/replicate and 22 rods/replicate). Basal loading in Ca^2+^-free, Cd^2+^-containing solution was significantly lower than loading for 10 min in control saline (unpaired *t*-tests: SR101: cones, *P* < 0.0001; rods, *P* = 0.0029. 3-kDa Texas Red: cones, *P* = 0.0128; rods, *P* = 0.0074. 10-kDa Texas Red: cones, *P* = 0.0031; rods, *P* = 0.0034. 70-kDa Texas Red: cones, *P* < 0.0001; rods, *P* = 0.015. pHrodo, cones, *P* = 0.0003; rods, *P* < 0.0001).

We began by studying the ability of isolated rods and cones to endocytose different dyes. After incubating isolated cells in dye for 3 or 10 min, fluorescent intensities of isolated rod and cone terminals were measured by epi-fluorescence. Uptake during this time was presumably due to endocytosis accompanying spontaneous vesicle release. Dye was concentrated in terminals as expected by uptake into synaptic vesicles (Figure 2B). Intraterminal fluorescence was significantly reduced (unpaired *t*-tests; see figure legend) by incubating rods and cones for 10 min with dye in the presence of Ca^2+^-free, high-Mg^2+^ solution containing 0.1 mM Cd^2+^ to block Ca^2+^ channels. This can be seen in the insets of Figure 2C showing measurements at low fluorescent intensities made after 10 min. Uptake of 3-kDa Texas Red after blocking Ca^2+^ channels was slightly greater than the other four dyes, but was still substantially lower than uptake in control conditions. This suggests a slightly greater propensity for uptake of 3-kDa Texas Red by Ca^2+^-independent vesicle cycling (Cork et al., 2016) or other mechanisms.

Fluid phase indicators enter vesicles during endocytosis by passive diffusion. Given an internal vesicle diameter of 30 nm and dye concentration of 7 μM, an average of 0.06 dye molecules should be present within an intra-vesicular volume of 1.4 × 10^−20^ L. Most vesicles should therefore be retrieved with either a single dye molecule or no dye. Consistent with this, single vesicle fluorescence measured by TIRFM after loading with 7 μM 70-kDa Texas Red (560.7 ± 334.9 [S.D.] fluorescence units, *N* = 168 vesicles) was not significantly (*P* = 0.063, F-test) greater than single 70-kDa Texas Red molecules (486.4 ± 166.9 units, *N* = 230). Thus, regardless of dye size, the overall intra-terminal concentrations of different dyes in this experiment should more closely reflect the number of dye-loaded vesicles than the concentration of dye within each vesicle.

Relative intra-terminal dye concentrations were calculated by dividing whole terminal fluorescence by the molar fluorescence of each dye measured *in vitro*. The fluorescence of pHrodo increases during vesicle re-acidification but terminal fluorescence was measured 3 and 10 min after loading, much longer than the time required for vesicle re-acidification (Gandhi and Stevens, 2003). Thus, like the other dyes, terminal fluorescence with pHrodo was determined by the rate of uptake, not re-acidification. The data fell into two distinct groups in both cones and rods: smaller dyes (0.6-kDa SR101 and 3-kDa Texas Red) were loaded equally well, whereas larger 10-kDa Texas Red, 10-kDa pHrodo, and 70-kDa Texas Red were loaded more poorly (Figure 2C). The differences between these two groups increased as the loading period was increased from 3 to 10 min. Preferential uptake of the small dyes over large dyes is consistent with the idea that small dyes can be retrieved by two modes—kiss-and-run and conventional endocytosis—whereas uptake of large dyes is restricted to conventional mechanisms of endocytosis.

### Dyes Were Loaded into Synaptic Vesicles

**Figure 3:**
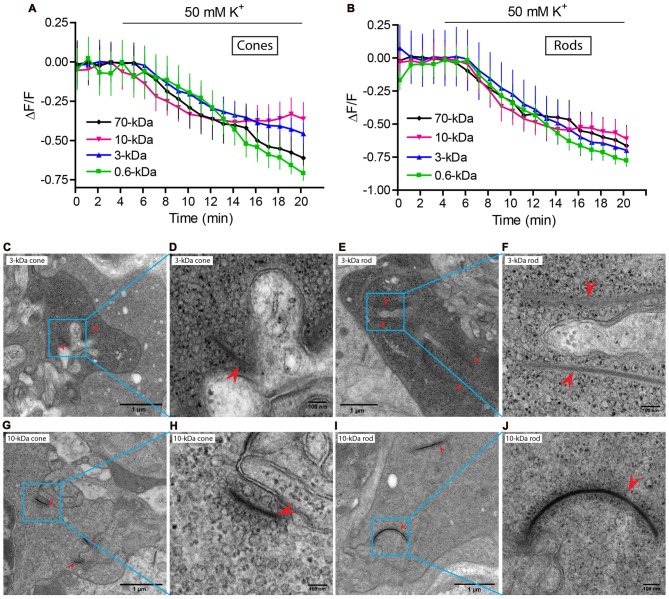
Dyes were loaded into synaptic vesicles. **(A,B)** Fluorescence (ΔF/F) of SR101, 3-, 10- and 70-kDa Texas Red declined upon application of 50 mM K^+^ in both cones **(A)** and rods **(B)**. Slices were incubated with SR 101 (83 μM, 30 min), 3-kDa Texas Red (133 μM, 45 min), 10-kDa Texas Red (100 μM, 60 min), and 70-kDa Texas Red (36 μM, 60 min). Each experiment was repeated three times and each data point shows the average ± SEM from 8 to 44 terminals. The capability of dyes to be released from retinal slices by depolarizing stimulation indicates they were loaded into releasable synaptic vesicles. **(C–J)** Electron micrographs of photoreceptor terminals loaded with lysine-fixable, 3-kDa Texas Red (7 μM). After photoconversion in the presence of DAB, electron dense precipitate can be seen in vesicles throughout the terminals. A cone terminal with its characteristically small ribbons (arrowheads) is shown in **(C)** and a rod terminal with its larger ribbons (arrowheads) in **(E)**. Panels **(D,F)** show magnified views of the square regions in **(C,E)**, respectively. **(G–J)** Electron micrograph of a photoreceptor terminal loaded with 10-kDa Texas Red (7 μM). **(G,I)** show a cone and a rod terminal, respectively. **(H,J)** show magnified views of the square regions in **(G,I)**.

To test whether dyes were loaded into synaptic vesicles, we incubated retinas with the various dyes and then measured their ability to be released by depolarizing stimulation with high KCl saline. For this experiment, we used higher dye concentrations and longer incubation times than the previous experiment (detailed in the legend for Figure 3). We found that all of the dyes (SR101, 3-kDa Texas Red, 10-kDa Texas Red, 70-kDa Texas Red and 10-kDa pHrodo) were released from rod and cone terminals by depolarizing stimulation with 50 mM K^+^ solution (Figures 3A,B) indicating that they were loaded into synaptic vesicles. We also confirmed that 3- and 10-kDa Texas Red could be released from terminals of isolated rods and cones by 50 mM K^+^ (data not shown).

As further confirmation that dyes were loaded into synaptic vesicles, we photoconverted 3- and 10-kDa Texas Red and examined their distribution within photoreceptor terminals by EM. We loaded retinal pieces with 3- or 10-kDa lysine-fixable Texas Red at a concentration of 7 μM for 2 min in Ca^2+^ free solution to allow dye levels to equilibrate throughout the retina and then stimulated vesicle turnover by incubating the retinal pieces in dye for another 8 min in 20 mM K^+^ solution. After fixation, we photoconverted dyes in the presence of DAB to generate an electron-opaque, osmiophilic precipitate for EM images (Maranto, 1982; Schmued and Snavely, 1993). Electron-dense particles were concentrated in synaptic vesicles of photoreceptor terminals loaded with either 3- or 10-kDa Texas Red (Figure 3). Photoreceptor terminals in the outer plexiform layer were identified by their electron-dense ribbons (arrowheads). Rods were distinguished from cones by their much longer ribbons (Lasansky, 1978). For both dyes, we illustrate an example of a rod and cone terminal at low and high magnification. Figures 3C,D show a cone terminal at low (C) and high (D) magnification after loading with 3-kDa Texas Red. Figures 3G,H show a cone terminal loaded with 10-kDa Texas Red. Figures 3E,F show a rod terminal loaded with 3-kDa Texas Red. Figures 3I,J show a rod terminal loaded with 10-kDa Texas Red. Electron-dense particles were observed in vesicles both near and far from synaptic ribbons consistent with previous studies suggesting mixing of vesicles throughout the cytoplasmic and ribbon pools (Ripps et al., 1976; Schacher et al., 1976; Schaeffer and Raviola, 1978; Townes-Anderson et al., 1985, 1988). Particles were heavily concentrated in photoreceptor terminals and rarely seen in other parts of photoreceptors or neighboring processes of horizontal and bipolar cells. As in an earlier study of endocytosis in salamander rods using horseradish peroxidase (Townes-Anderson et al., 1985), electron-dense particles were occasionally observed in larger vacuoles, but the vast majority were located in synaptic vesicles. These experiments confirmed that the dyes used in our experiments are endocytosed into synaptic vesicles. In addition, more vesicles appeared to contain particles in terminals loaded with 3-kDa Texas Red than in terminals loaded with 10-kDa Texas Red (Figures 3C–J), consistent with preferential endocytosis of smaller molecules.

### Impact of Endocytosis Inhibitors on Uptake of Large and Small Dyes

**Figure 4:**
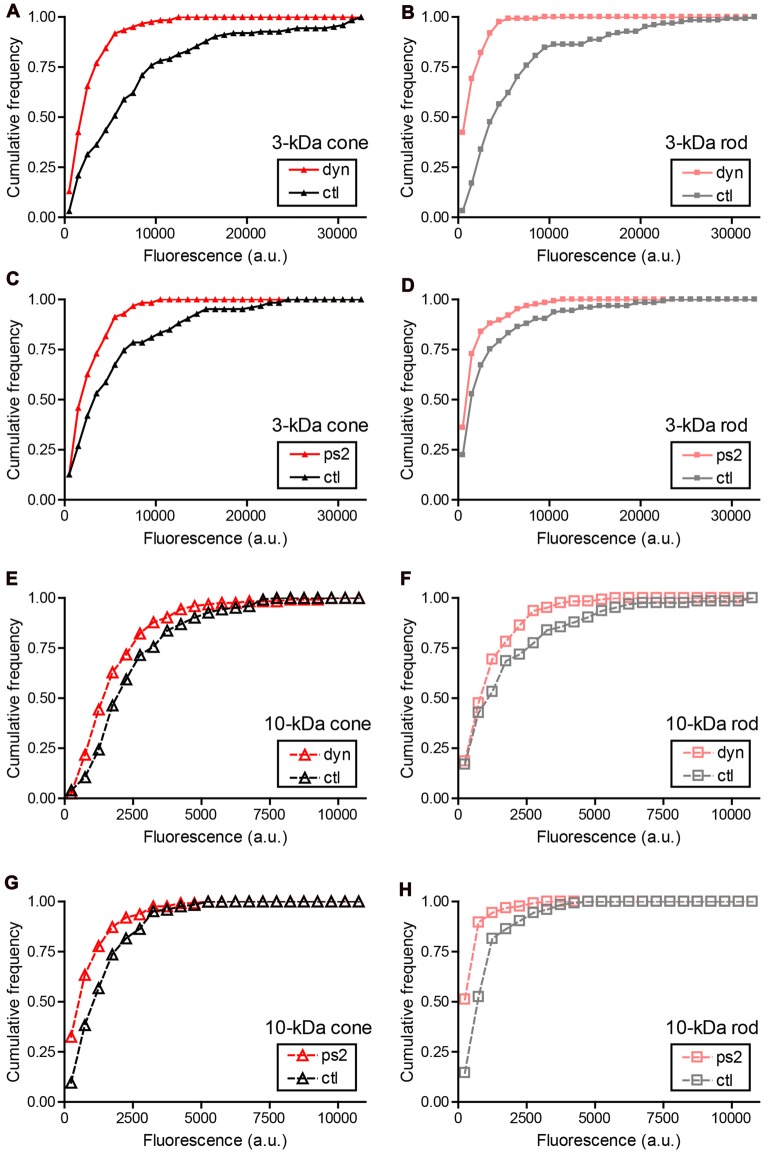
Sensitivity of small and large dyes to endocytic inhibitors. Cumulative frequency histograms of whole-terminal fluorescence measured in rods and cones after incubation with 3-kDa or 10-kDa Texas Red. Retinas were incubated for 20 min in a test solution (dynasore, pitstop-2, or vehicle control) and then another 10 min in test solution supplemented with 3-kDa (67 μM) or 10-kDa Texas Red (50 μM). Retinal cells were then isolated and plated onto coverslips. Data in each condition were obtained from multiple cells in three replicate experiments. **(A)** Cones loaded with 3-kDa Texas Red and treated with dynasore (80 μM, *N* = 122 cells) or vehicle control (0.1% DMSO, *N* = 124). The cumulative distributions of intraterminal fluorescence differed significantly by a Kolmogorov–Smirnov test (*P* < 0.0001). **(B)** Rods loaded with 3-kDa Texas Red and treated with dynasore (*N* = 123) or vehicle control (*N* = 124; *P* < 0.0001, Kolmogorov–Smirnov test). **(C)** Cones loaded with 3-kDa Texas Red and treated with pitstop-2 (25 μM, *N* = 126) or vehicle control (0.2% DMSO, *N* = 126; *P* < 0.0001). **(D)** Rods loaded with 3-kDa Texas Red and treated with pitstop-2 (*N* = 125) or vehicle control (*N* = 125; *P* = 0.002). **(E)** Cones loaded with 10-kDa Texas Red and treated with dynasore (*N* = 124) or vehicle control (*N* = 123; *P* < 0.0001). **(F)** Rods loaded with 10-kDa Texas Red and treated with dynasore (*N* = 124) or vehicle control (*N* = 124; *P* = 0.035). **(G)** Cones loaded with 10-kDa Texas Red and treated with pitstop-2 (*N* = 126) or vehicle control (*N* = 125; *P* < 0.0001). **(H)** Rods loaded with 10-kDa Texas Red and treated with pitstop-2 (*N* = 125) or vehicle control (*N* = 124; *P* < 0.0001).

Different modes of synaptic endocytosis depend differently on clathrin and dynamin in different cells (Artalejo et al., 1995; Granseth et al., 2006; Ferguson et al., 2007; Watanabe et al., 2014; Kononenko and Haucke, 2015). Uptake of 3-kDa Texas Red into cone and rod terminals was significantly inhibited (see Figure 4 legend for statistics) by the dynamin inhibitor, dynasore (80 μM), and clathrin inhibitor, pitstop-2 (25 μM), with dynasore producing somewhat greater inhibition than pitstop-2. Uptake of 10-kDa Texas Red was inhibited to a similar extent by both dynasore and pitstop-2 (Figures 4E–H). A larger fraction of 3-kDa Texas Red uptake into rod and cone terminals was inhibited by dynasore treatment than 10-kDa Texas Red uptake (Figure 4). This may be partly due to the finding in Figure 2 that a larger fraction of the total uptake of 3-kDa Texas Red appears to be synaptic in origin—and is thus more likely to be sensitive to dynasore—than uptake of 10-kDa Texas Red.

### Comparisons of Endocytosis in Individual Vesicles

Whole-terminal fluorescence measurements and ultrastructural images both indicated that a larger number of vesicles endocytosed small dyes than large dyes when exposed to similar concentrations. To compare the ability of vesicles to take up large and small dyes more directly, we used TIRFM techniques (Chen et al., 2013) to examine uptake into individual vesicles that were incubated simultaneously in 10-kDa and 3-kDa dyes.

**Figure 5:**
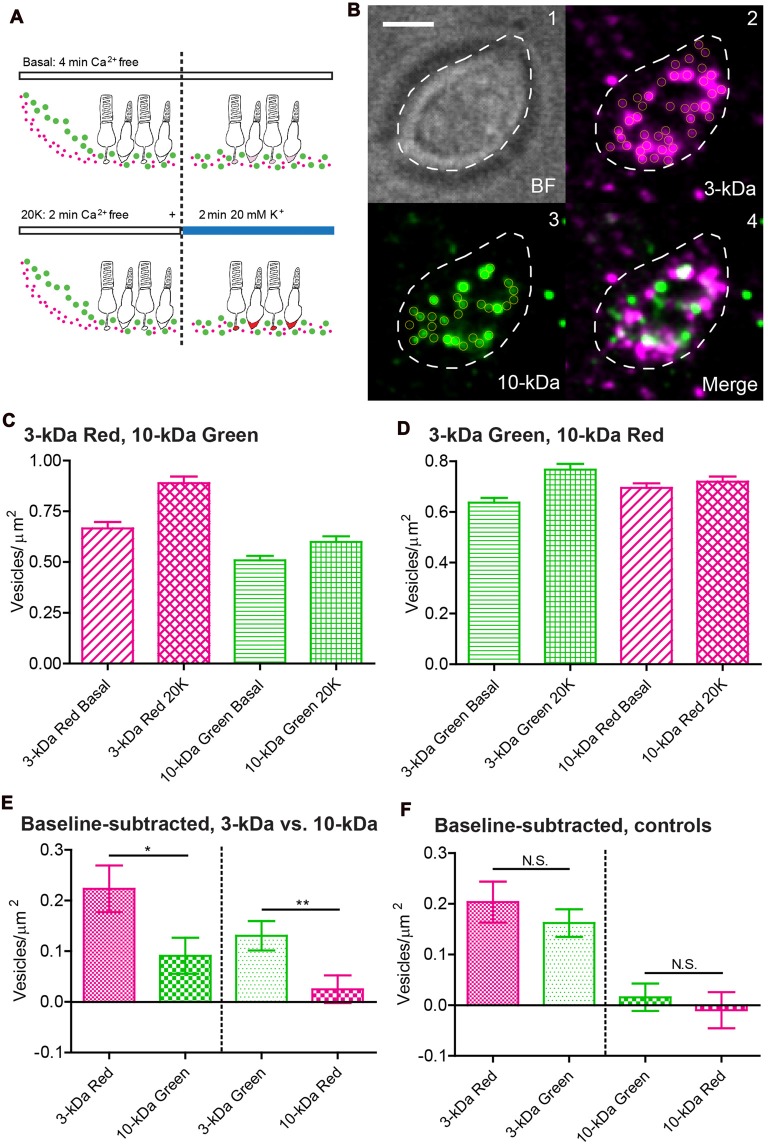
By incubating rods simultaneously in red and green dyes, total internal reflection fluorescence microscopy (TIRFM) imaging of individual synaptic vesicles showed that more vesicles were loaded with small dyes than large dyes. **(A)** Loading protocol: We incubated retinal pieces for 2 min in Ca^2+^-free, high-Mg^2+^ saline to equilibrate dye throughout the retina. To measure basal loading, we maintained retinas for another 2 min in Ca^2+^-free saline. To measure depolarization-evoked loading, we incubated retinas for the final 2 min in 20 mM K^+^/1.8 mM Ca^2+^. **(B1)** Bright-field image of an isolated rod terminal. **(B2)** Average TIRFM image (30 × 40 ms/frame, 140-ms intervals) of the same terminal showed many fluorescent vesicles (yellow circles) after incubation with 3-kDa Texas Red. Vesicle brightness in these average images was largely a function of the time each vesicle spent in the evanescent field near the membrane during the trial. **(B3)** Simultaneous incubation with 10-kDa AlexaFluor-488 loaded fewer vesicles. **(B4)** Merged image of 3-kDa Texas Red (magenta) and 10-kDa AlexFluor-488 (green) dyes. Scale bar: 2.5 μm. **(C,D)** Bar graphs show vesicles/μm^2^ observed using TIRFM under basal Ca^2+^-free and 20 mM high-K^+^ conditions. 3-kDa Texas Red vs. 10-kDa AlexaFluor-488 in (**C**; basal: *N* = 83 terminals; 20-K^+^: *N* = 108); 3-kDa AlexaFluor-488 vs. 10-kDa Texas Red in (**D**; basal: *N* = 132; 20-K^+^: *N* = 109). **(E,F)** Bar graphs show vesicles/μm^2^ observed with high-K^+^ stimulation after subtracting basal loading. Panel **(E)** compares loading of 3-kDa vs. 10-kDa dyes. Panel **(F)** shows control experiments comparing loading of 3-kDa Texas Red vs. 3-kDa AlexFluor-488 (basal: *N* = 132; 20-K^+^: *N* = 116) and 10-kDa Texas Red vs. 10-kDa AlexFluor-488 dyes (basal: *N* = 113; 20-K^+^: *N* = 122). *P < 0.05, **P < 0.01, N.S. *P* > 0.05 (*t*-test).

We first incubated rods simultaneously with both red 3-kDa Texas Red and green 10-kDa AlexaFluor-488 (Figure 5A). Salamander rods possess relatively large synaptic terminals that, when flattened onto the coverslip, can produce a region of membrane contact that is sufficiently large for TIRFM imaging. A faint fluorescent footprint reveals the region of contact between the coverslip and synaptic terminal membrane in the average images. We used rods for these experiments because cones rarely exhibited synaptic terminal footprints that were suitable for TIRFM imaging. Individual bright spots could be seen in this footprint region (Figure 5B), representing dye-loaded vesicles that advanced to the membrane and entered the evanescent field of illumination during the acquisition period. The evanescent wave in TIRFM decays with a length constants of ~60 nm, somewhat longer than the diameter of a single vesicle. The x-y resolution has a full-width half maximum of ~350 nm, but the incubation protocol loads only a small percentage of vesicles and so most individual bright spots were likely to represent the fluorescence of single vesicles (Chen et al., 2013). As discussed above, the low dye concentration (7 μM) and small intravesicular volume limited the likelihood that two dye particles were loaded into the same vesicle. We interleaved 488 and 565 nm excitation (30 frames apiece at 40 ms/frame and 140-ms intervals). In average images from this sequence (e.g., Figure 5B), the brightness of a fluorescent spot was largely a function of the residence time of a dye-loaded vesicle at the membrane rather than the concentration of dye in each individual vesicle. The low percentage of loaded vesicles explains the limited overlap of red and green fluorescence in the average image (Figure 5B).

We collected a single trial for each rod terminal and identified local maxima in the average image (yellow circles in Figure 5B) to count the number of vesicles loaded with red and green dyes. We typically saw 20–30 dye-labeled vesicles per terminal footprint (33.5 ± 0.5 μm^2^, *N* = 914 terminals). To eliminate differences in loading that could arise from differences in the rate of dye diffusion through the retina, we began both control and test trials by loading the retina for 2 min in Ca^2+^-free saline (Figure 5A). In control trials, we then kept retinas in Ca^2+^-free saline for another 2 min (for a total of 4 min). In test trials, after the first 2 min in Ca^2+^-free saline, we incubated retinas for another 2 min in 20 mM K^+^ saline with 1.8 mM Ca^2+^ to stimulate vesicle cycling and measure the attendant synaptic endocytosis. The number of vesicles loaded with 3-kDa Texas Red averaged 0.67 ± 0.29 vesicles/μm^2^ (*N* = 83 terminals) in basal conditions and 0.89 ± 0.34 vesicles/μm^2^ (*N* = 108) following high-K^+^ stimulation (Figure 5C). Rods are capable of considerable Ca^2+^-independent spontaneous release that can account for much of the loading under basal, Ca^2+^-free conditions (Cork et al., 2016). After subtracting basal loading, the net high-K^+^-evoked increase in endocytosis averaged 0.22 ± 0.05 vesicles/μm^2^ with 3-kDa Texas Red. Significantly fewer vesicles were loaded with 10-kDa AlexaFluor-488 in the same synaptic terminals, with a net high-K^+^-evoked increase in loading of 0.09 ± 0.04 vesicles/μm^2^ (*P* = 0.024, unpaired *t*-test). Although the smaller 3-kDa dye has fewer fluorophore moieties attached to each dextran molecule, more vesicles were loaded with 3-kDa dye (Figure 5B) and so we observed greater background fluorescence with this dye than with 10-kDa dye. This worsened the signal-to-noise ratio and so we may have under-counted the number of vesicles loaded with 3-kDa dyes. To control for differences in loading or detection arising from properties of the fluorophore other than molecule size, we swapped the fluorophores to measure endocytosis into vesicles that were incubated simultaneously in 10-kDa Texas Red and 3-kDa AlexaFluor-488 (Figure 5D). As with the previous comparison of small red dye vs. large green dye, the net high K^+^-evoked increase in vesicles loaded with the small green dye, 3-kDa AlexaFluor-488, was significantly greater than the increase in vesicles loaded with large 10-kDa Texas Red (*N* = 109, *P* = 0.0085; Figure 5E). As additional controls, we compared loading of 3-kDa Texas Red with 3-kDa AlexaFluor-488 and 10-kDa Texas Red with 10-kDa AlexaFluor-488 (Figure 5F). We found no significant differences in net loading between red and green dyes of the same size (3-kDa, *N* = 116, *P* = 0.40; 10-kDa, *N* = 122, *P* = 0.57). Thus, consistent with whole-terminal fluorescence measurements of endocytosis and EM experiments, these data show that individual vesicles take up 2.3 nm 3-kDa dyes more frequently than 4.6 nm 10-kDa dyes.

### Two Modes of Synaptic Vesicle Exocytosis

The greater uptake of small dyes compared to large dyes suggests there are at least two modes of endocytosis in photoreceptors, with one mode favoring uptake of small molecules with diameter <4.6 nm. If the endocytic mode that preferentially takes up small molecules involves kiss-and-run, then we should also see differences in exocytosis since the processes of release and re-uptake are intrinsically coupled to one another during kiss-and-run. While full-collapse fusion should release large and small molecules equally, kiss-and-run should be incapable of releasing molecules that are larger than the fusion pore (Figure 7A).

**Figure 6:**
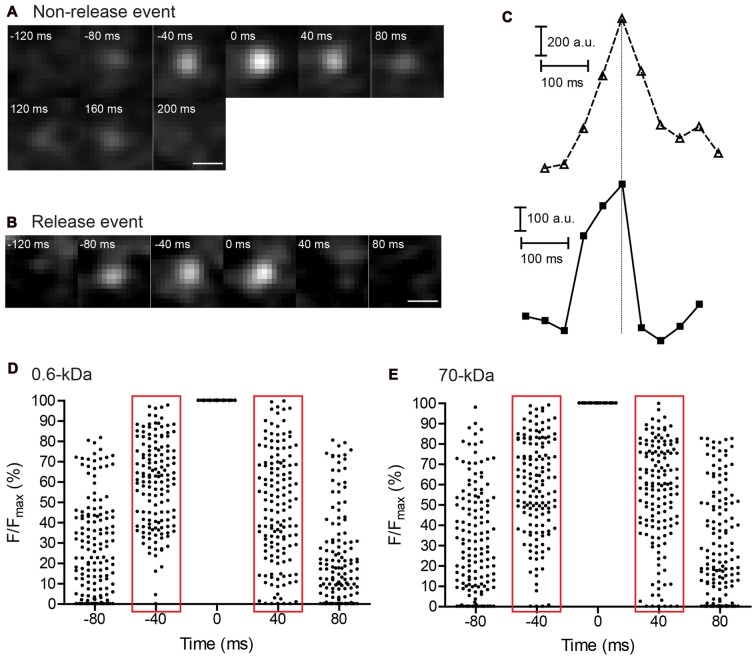
Imaging of individual vesicle release events using TIRFM in rods. Consecutive 40-ms images showing a non-release event **(A)** and release event **(B)** in individual synaptic vesicles loaded with SR101. Scale bars: 0.5 μm. **(C)** Fluorescence changes were measured within a 7 × 7 pixel region of interest enclosing the vesicles in (**A**; top) and (**B**; bottom) and plotted as a function of time. Vertical line aligns the peak fluorescence values. Vesicle fluorescence declined more rapidly during release than during non-release events in which fluorescence declined at a rate similar to the rate of increase observed as a vesicle approached the membrane. **(D,E)** Scatter plots of normalized fluorescence values measured in individual vesicles during the final two 40-ms frames of vesicle approach to the membrane and the first two frames during fluorescence decline. Data show vesicles loaded with SR101 (*N* = 143 events) **(D)** and 70-kDa Texas Red (*N* = 142) **(E)** during 50 mM K^+^ puff.

**Figure 7:**
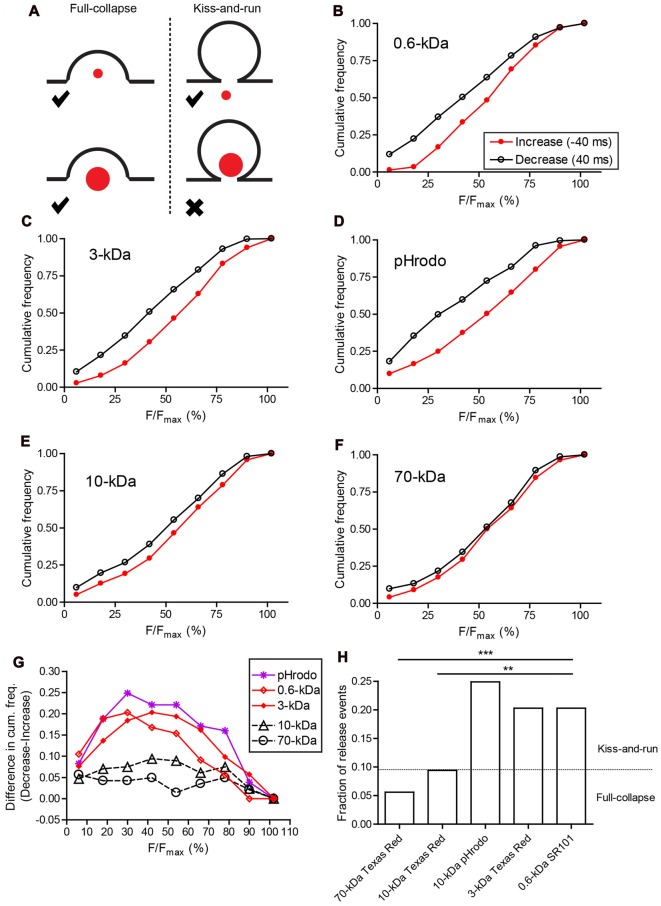
Two modes of synaptic vesicle exocytosis. **(A)** Schematic image illustrating that small dyes can be released by both full-collapse and kiss-and-run exocytosis, whereas large dyes can only be released by full-collapse. **(B–F)** Cumulative histograms of relative fluorescence values measured in individual vesicles one 40-ms frame before the peak (increase; red filled circles) and one frame after peak fluorescence was attained (decrease; black open circles). **(G)** Differences in cumulative frequency between fluorescence decrease and increase from **(B–F)** used to assess the fraction of events due to release. **(H)** Bar graph of release fractions determined from the maximum difference in cumulative frequency for each dye in **(G)**. Fractions of release events seen with SR101 (*N* = 143 events), 3-kDa Texas Red (*N* = 315), and pHrodo (*N* = 181) were significantly greater than fractions of release events seen with 10-kDa (*N* = 213) and 70-kDa Texas Red (*N* = 142). **P < 0.01, ***P < 0.001 (*z*-test: pHrodo vs. 10-kDa Texas Red: *P* < 0.0001; pHrodo vs. 70-kDa Texas Red: *P* < 0.0001; SR101 vs. 10-kDa Texas Red: *P* = 0.0034; SR101 vs. 70-kDa Texas Red: *P* = 0.0002; 3-kDa Texas Red vs. 10-kDa Texas Red: *P* = 0.0008; 3-kDa Texas Red vs. 70-kDa Texas Red: *P* < 0.0001).

We examined release of various dyes by studying exocytosis of individual vesicles in rods using TIRFM (Chen et al., 2013). As a vesicle approaches the membrane, it becomes progressively brighter at a rate determined by its advance through the exponentially increasing evanescent field intensity (Figures 6A,B). If, after contacting the membrane, a vesicle retreats from the membrane without fusion, then it should decline in brightness at a rate paralleling the rate at which vesicle brightness increases during approach (Figure 6A). Figure 6A shows a non-release event for a vesicle loaded with SR101 in which vesicle fluorescence increased in a roughly exponential fashion as it approached the membrane and then decreased at a similar rate as it departed the membrane. If on the other hand, a vesicle releases its contents, then its fluorescence should decline almost instantly, as illustrated by the example in Figures 6B,C. Assuming a diffusion coefficient in water of 5.8 × 10^−7^ cm^2^/s, a single 70-kDa Texas Red molecule should diffuse an average of 2.2 μm (27 pixels) within a single 40-ms frame and therefore disappear from view almost immediately after release. For TIRFM experiments on release, we used higher dye concentrations than for endocytosis experiments: SR101 (8.3 μM), 3-kDa Texas Red (133.3 μM), 10-kDa Texas Red (50 μM), pHrodo (50 μM) and 70-kDa Texas Red (14.3 μM). This increased the likelihood that a vesicle may contain more than a single dye molecule. By selecting the brightest vesicles for imaging, we also may have preferentially selected vesicles from the sub-population that contained more than a single dye molecule.

From differences in the kinetics of appearance and disappearance, we distinguished release events from non-release events where vesicles departed the membrane without fusion. Figures 6D,E show scatter plots of relative fluorescence intensities (normalized to peak fluorescence) measured in individual vesicles during depolarizing stimulation with a puff of 50 mM K^+^. We illustrate fluorescence changes in vesicles loaded with 0.6-kDa SR101 (Figure 6D) and 70-kDa Texas Red (Figure 6E). With both dyes, vesicle brightness increased gradually during the final two 40-ms frames of membrane before attaining peak fluorescence (Figure 6). The relative fluorescence of a vesicle as it approaches the membrane can be converted to vesicle position within the submembrane evanescent field and then used to calculate approach velocity. Approach velocities calculated from vesicle position in the penultimate frame during membrane approach were similar (*P* = 0.30; Student’s *t*-test) for both SR101 (537 ± 40 nm/s, *N* = 143 events) and 70-kDa Texas Red (612 ± 60 nm/s, *N* = 142) and similar to earlier measurements made with other dyes (Zenisek et al., 2000; Chen et al., 2013). After reaching the membrane, vesicle fluorescence typically remained elevated for a short time and then declined in intensity (Chen et al., 2013). When analyzing vesicles that remained stably associated with the membrane for two or more frames, we measured the increase in fluorescence relative to the initial peak attained during approach and measured the decline in fluorescence relative to the final peak intensity just before it began to fall. For vesicles loaded with 70-kDa Texas Red (Figure 6E), fluorescence intensity generally declined at a similar rate as it rose during membrane approach suggesting most of these vesicles disappeared because of membrane departure, not fusion. Thus, for vesicles loaded with 70-kDa Texas Red, apparent velocities calculated from the rate of vesicle disappearance in one frame (736 ± 74 nm/s, *N* = 142) did not differ significantly from approach velocities (*P* = 0.19, unpaired *t*-test). Similar approach and departure kinetics for non-release events was also seen with pHrodo-loaded vesicles by Chen et al. (2013) and may reflect high vesicle mobility at photoreceptor ribbon synapses (Rea et al., 2004). Many more of the vesicles loaded with the small dye, SR101 (Figure 6D), showed rapid fluorescence declines. For example, during membrane approach, only 5 of 143 SR101-loaded vesicles exhibited an intensity <25% of its peak value by the penultimate frame. By contrast, six times as many vesicles (32) had fallen to <25% peak fluorescence by the first frame during the decline phase. Because of the greater number of release events in which fluorescence declined precipitously, the average apparent velocity calculated from the decline in fluorescence of SR101-loaded vesicles by the first frame after the peak (928 ± 80 nm/s, *N* = 143 events) was significantly faster than the approach velocity calculated from the rise in the fluorescence during the final frame (*P* < 0.0001).

To assess the frequency of release events, we compared cumulative frequency histograms of relative fluorescence changes during the final 40-ms frame of membrane approach and the first frame of the decline phase (Figures 6D,E). For 70-kDa Texas Red, the two histograms were nearly identical indicating that rates of vesicle appearance and disappearance were very similar and therefore that most disappearance events were due to membrane departure rather than fusion (Figure 7F). Histograms for 10-kDa Texas Red were also similar to one another (Figure 7E). However, with SR101, 3-kDa Texas Red, and 10-kDa pHrodo, there were noticeably larger differences between the histograms of fluorescence increase and decrease (Figures 7B–D), suggesting many more release events. To determine the fraction of vesicles that disappeared more rapidly than they appeared during approach, we calculated differences between the cumulative histograms for fluorescence increase and decrease with each dye (Figure 7G). From the peak differences between these cumulative histograms, we determined that 20% of the vesicles loaded with SR101 (*N* = 143 events) or 3-kDa Texas Red (*N* = 315) disappeared more rapidly than they appeared and were thus identified as release events (Figures 7G,H). Twenty-five percent of pHrodo-loaded vesicles were classified as release events in this way. By contrast, for 70-kDa (*N* = 142) and 10-kDa Texas Red (*N* = 213), only 6%–9% of the events showed faster disappearance and were categorized as release events (Figures 7G,H). Thus, similar to uptake experiments, release fell into two distinct groups with 10-kDa and 70-kDa Texas Red showing significantly less release than the other dyes (see figure legend for statistics).

The use of 10-kDa pHrodo provided a useful test of the hypothesis that differences in uptake and release between large and small molecules involve significant contributions from kiss-and-run. Recall that in uptake experiments, 10-kDa pHrodo was endocytosed poorly like 10-kDa and 70-kDa Texas Red. On the other hand, pHrodo behaved like smaller dyes in release experiments. What can explain this difference? Unlike Texas Red, pHrodo fluoresces more brightly in the acidic environment of the vesicle interior and is quenched by alkaline extracellular pH^2^. Thus, while 10-kDa pHrodo itself may be retained within a vesicle during kiss-and-run, protons can readily exit the vesicle through a small fusion pore, causing pHrodo fluorescence to decline abruptly. An abrupt decline in fluorescence would classify this as a fusion event. The finding that the percentage of pHrodo-loaded vesicles classified as release events was similar to the percentage of release events for SR101 and 3-kDa Texas Red supports the conclusion that kiss-and-run contributes to a large fraction of release events. If large molecules (10-kDa and 70-kDa Texas Red) can only be released by full-collapse whereas small molecules (SR101, 3-kDa Texas Red, and intravesicular protons) can be released by both full-collapse and kiss-and-run, then these data indicate that full-collapse contributes to 24%–45% of release events identified by our criteria (6%–9%/20%–25%) and kiss-and-run contributes to the remaining 55%–66% (Figure 7H).

## Discussion

### Kiss-and-Run Is Favored in Photoreceptors

A key property of kiss-and-run is the transient opening of a small fusion pore that allows release of vesicle contents without complete fusion with the plasma membrane. We found that molecules ≤2.3 nm diameter were released and retrieved much more readily than molecules ≥4.6 nm. This suggests there are two modes of release and re-uptake: one involving kiss-and-run fusion with a pore diameter of 2.3–4.6 nm and a second involving full-collapse followed by conventional endocytosis in which large and small dyes are released and retrieved equally well. We found that 10-kDa pHrodo loaded poorly like 10-kDa and 70-kDa Texas Red, but exhibited release properties similar to smaller dyes in TIRFM release experiments. This can be explained by the pH-dependence of pHrodo in which departure of intravesicular protons through a fusion pore during kiss-and-run can rapidly quench pHrodo fluorescence even while the dye itself is retained within the vesicle. Because loading of 10-kDa pHrodo likely occurs by conventional endocytic mechanisms, this suggests that vesicles retrieved by conventional endocytosis can subsequently participate in kiss-and-run release. Consistent with a mixing of vesicles after retrieval and with earlier studies (Ripps et al., 1976; Schacher et al., 1976; Schaeffer and Raviola, 1978; Townes-Anderson et al., 1985, 1988), photoconversion experiments showed that vesicles loaded with both 3- and 10-kDa Texas Red were dispersed throughout the terminal.

CNS neurons can retrieve synaptic vesicles by fast and slow forms of endocytosis (von Gersdorff and Matthews, 1994; Jockusch et al., 2005; Van Hook and Thoreson, 2012; Watanabe et al., 2013; Delvendahl et al., 2016; Liang et al., 2017; Watanabe and Boucrot, 2017). While there are exceptions, vesicle fission typically requires the GTPase dynamin. Slower forms of endocytosis also typically involve formation of a clathrin protein lattice whereas faster forms can proceed without clathrin (Soykan et al., 2016). We observed inhibition of uptake with dynasore and pitstop-2 indicating the presence of both dynamin- and clathrin-dependent retrieval processes. Measurements of membrane capacitance in rods and cones suggested the presence of both fast and slow forms of endocytosis with the rapid form (τ ~600 ms) accounting for at least 70% of membrane retrieval. Although other forms of endocytosis can also produce rapid declines in membrane capacitance (Elhamdani et al., 2006; He et al., 2006; Wu and Wu, 2007; Watanabe and Boucrot, 2017), the initial rapid decline in capacitance that we observed is consistent with TIRFM results suggesting that kiss-and-run contributes to a majority of release events.

To identify release events using TIRFM, we developed stringent criteria to exclude events in which vesicle disappearance might be due to retreat from the membrane without fusion. Because we compared release event ratios and not absolute numbers, the larger percentage of release events observed with SR101, 3-kDa Texas Red, and pHrodo in TIRFM experiments was not a result of differences in dye loading. Furthermore, pHrodo exhibited a similar release percentage to smaller dyes even though it was endocytosed much more poorly. All vesicles analyzed for TIRFM release experiments showed similar signal-to-noise properties and so noise-related errors should be similar among dyes. Differences in diffusion kinetics among dyes were not large enough to significantly impact rates of vesicle disappearance. For example, 3-kDa and 10-kDa dye particles should depart a 7 × 7 pixel region of interest in 0.88 and 1.36 ms, respectively, creating only 0.6% difference in probability of detecting release by our criteria. It is therefore unlikely that the criteria used to identify release events selectively favored detection of release when using SR101, 3-kDa Texas Red, or pHrodo.

While the most parsimonious explanation for our data involves two modes of release, full-collapse and kiss-and-run, we also considered alternatives that might require only full-collapse. A possible alternative explanation for preferential detection of release with small molecules that involves only full-collapse could be that the fusion pore dilates very slowly to allow release of small molecules many milliseconds before release of larger molecules. While such a mechanism could promote faster disappearance of vesicles loaded with small dyes, it should also cause a graded increase in the likelihood of detecting release events with decreasing molecular size. Instead, we found that release clustered into two distinct groups. This hypothetical full-collapse mechanism would also have to incorporate preferential uptake of small dyes during endocytosis. This could be achieved if, during endocytic retrieval of vesicles after full collapse, the vesicle lumen remained in contact with the extracellular space through a narrow neck for an extended time prior to fission. However, once again, this mechanism would predict a graded increase in uptake efficiency with decreasing molecular size, not the two distinct groups that we observed. This mechanism also predicts that increasing the persistence of vesicles at the membrane should increase the preferential uptake of small dyes. An increase in the persistence of hemifused vesicles at the membrane can be achieved by inhibiting dynamin GTPase activity (Newton et al., 2006; Logiudice et al., 2009). However, contrary to this prediction, we found that inhibiting dynamin with dynasore reduced uptake of small dyes rather than promoting their preferential uptake. Thus, the preferential release and re-uptake of small molecules cannot be explained by a single full-collapse mechanism and is instead more likely due to significant contributions from kiss-and-run.

Consistent with our findings, capacitance measurements from large secretory vesicles in neuroendocrine cells (Spruce et al., 1990; Albillos et al., 1997; Fulop et al., 2005) and small synaptic vesicles in neurons (He et al., 2006) have found that fusion pores formed during the first few milliseconds of fusion or during stand-alone kiss-and-run events can have a diameter of 1–3 nm. On the other hand, fusion pores of 0.3 nm diameter or smaller have been observed in synaptic-like microvesicles of pituitary cells (Klyachko and Jackson, 2002) and small secretory granules of neutrophils (Lollike et al., 1995, 1998). Lollike et al. (1998) suggested that further dilation of a pore beyond its initial small size may lead irreversibly to fusion of small secretory granules. On the other hand, imaging of large dense core granules in chromaffin cells suggests that the fusion pore connecting a hemifused vesicle to the plasma membrane may never fully dilate (Chiang et al., 2014; Zhao et al., 2016). Electron tomography has shown omega figures characteristic of hemifused vesicles at rod synapses in mouse retina (Zampighi et al., 2011).

### Functional Significance for Retinal Processing and Vision

Cones release vesicles exclusively from ribbons (Snellman et al., 2011; Van Hook and Thoreson, 2015) at a steady rate in darkness of ~2 vesicles/s per ribbon release site (Heidelberger et al., 2005). Rods release vesicles at slower rates, with release occurring at both ribbon and non-ribbon release sites (Sheng et al., 2007; Babai et al., 2010; Chen et al., 2013). We only examined release with TIRFM techniques in rods, but the similar capacitance responses and behavior in endocytosis assays of rods and cones suggests that retrieval in both cell types likely involves kiss-and-run. One advantage of kiss-and-run is that it provides a mechanism to return synaptic vesicles rapidly to the releasable pool. However, re-loading of vesicles with glutamate is slow, with a time constant of ~15 s (Hori and Takahashi, 2012), and so rapid endocytosis may not greatly speed the return of vesicles to a fully functional status. Instead, the fast rate of vesicle retrieval in photoreceptors may be more important for clearing used synaptic proteins and lipids from the active zone to restore release site function (Hosoi et al., 2009; Hua et al., 2013; Lipstein et al., 2013; Mahapatra et al., 2016). This role for endocytosis may be particularly important in photoreceptors where release occurs continuously at numerous closely adjacent sites along the base of the ribbon. Another potential advantage of kiss-and-run is that it could limit changes to the active zone membrane that might otherwise be caused by the merger of vesicle membrane proteins and lipids during full-collapse fusion.

What are the post-synaptic effects of kiss-and-run release? The 10%–90% rise time of miniature excitatory postsynaptic currents (mEPSCs) at rod and cone synapses averages ~0.7 ms (Cadetti et al., 2008; Feigenspan and Babai, 2015; Cork et al., 2016), slower than many synapses. Synaptic cleft glutamate levels reach ~1 mM (Kim and Miller, 1993; Cadetti et al., 2008). Modeling suggests that glutamate released through a 2-nm pore can attain a concentration of >3 mM at a distance of 20 nm within 0.7 ms (Jackson, 2007). Thus, glutamate release through kiss-and-run pores can account for properties of mEPSCs observed in second-order retinal neurons.

Release at photoreceptor ribbon synapses involves a number of specialized synaptic proteins. In addition to the ribbon protein RIBEYE (Schmitz et al., 2000), other specialized proteins include syntaxin 3b (Morgans et al., 1996; Curtis et al., 2010) and complexins 3 and 4 (Reim et al., 2005). The Ca^2+^ sensor that mediates release from photoreceptors has not been identified but shows a higher Ca^2+^ affinity and lower cooperativity than synaptotagmin 1 (Thoreson et al., 2004; Duncan et al., 2010). Syntaxin, complexin and synaptotagmin have all been implicated in regulating fusion pore size and duration (Archer et al., 2002; Wang et al., 2003; Han et al., 2004; Zhang et al., 2011; Dhara et al., 2014; Rao et al., 2017). The protein isoforms employed at photoreceptor synapses may favor kiss-and-run, although a number of these isoforms are also present at bipolar cell ribbon synapses where kiss-and-run appears to be rare (Zenisek et al., 2002). Identifying the particular specializations that promote kiss-and-run in photoreceptors may help to identify factors regulating release mode in other CNS neurons.

The amount of kiss-and-run release found at CNS synapses varies among preparations and even between studies using the same preparation. Studies in retinal bipolar cells, hippocampal neurons, and the calyx of Held suggest that kiss-and-run is a relatively rare occurrence (Zenisek et al., 2002; Granseth et al., 2006; He et al., 2006; Balaji and Ryan, 2007; Chen et al., 2008). However, other studies from hippocampal neurons (Gandhi and Stevens, 2003; Richards et al., 2005; Zhang et al., 2009) and studies in lamprey reticulospinal synapses (Photowala et al., 2006) suggest that it can sometimes be quite common. The preferential uptake and release of small molecules, along with the rapid kinetics of endocytic retrieval, suggest that kiss-and-run mechanisms contribute significantly to vesicle cycling at photoreceptor synapses. This efficient mode of retrieval may promote rapid recycling that is particularly important at photoreceptor ribbon synapses to maintain the structure and function of release sites during continuous release.

## Author Contributions

XW and WBT designed and performed experiments, analyzed data and wrote the article together; GWS performed experiments, analyzed data and edited the article.

## Conflict of Interest Statement

The authors declare that the research was conducted in the absence of any commercial or financial relationships that could be construed as a potential conflict of interest.

## Footnotes

^1^https://www.sigmaaldrich.com/content/dam/sigma-aldrich/docs/Sigma/Product_Information_Sheet/1/fd250spis.pdf

^2^https://www.thermofisher.com/us/en/home/brands/molecular-probes/key-molecular-probes-products/phrodo-indicators.html
